# Impact of coadministration of apigenin and bone marrow stromal cells on damaged ovaries due to chemotherapy in rat: An experimental study

**DOI:** 10.18502/ijrm.v13i7.7372

**Published:** 2020-07-22

**Authors:** Athar Talebi, Nasim Hayati Roodbari, Hamid Reza Sameni, Sam Zarbakhsh

**Affiliations:** ^1^Department of Biology, Science and Research Branch, Islamic Azad University, Tehran, Iran.; ^2^Nervous System Stem Cells Research Center, Semnan University of Medical Sciences, Semnan, Iran.

**Keywords:** Apigenin, Bone marrow stromal cells, Chemotherapy, Ovary, Regeneration.

## Abstract

**Background:**

Apigenin is a plant-derived flavonoid with antioxidative and antiapoptotic effects. Bone marrow stromal cells (BMSCs) are a type of mesenchymal stem cells (MSCs) that may recover damaged ovaries. It seems that apigenin may promote the differentiation of MSCs.

**Objective:**

The aim of this study was to investigate the effect of coadministration of apigenin and BMSCs on the function, structure, and apoptosis of the damaged ovaries after creating a chemotherapy model with cyclophosphamide in rat.

**Materials and Methods:**

For chemotherapy induction and ovary destruction, cyclophosphamide was injected intraperitoneally to 40 female Wistar rats (weighing 180–200 gr, 10 wk old) for 14 days. Then, the rats were randomly divided into four groups (n = 10/each): control, apigenin, BMSCs and coadministration of apigenin and BMSCs. Injection of apigenin was performed intraperitoneally and BMSC transplantation was performed locally in the ovaries. The level of anti-mullerian hormone serum by ELISA kit, the number of oocytes by superovulation, the number of ovarian follicles in different stages by H&E staining, and the expression of ovarian Bcl-2 and Bax proteins by western blot were assessed after four wk.

**Results:**

The results of serum anti-mullerian hormone level, number of oocytes and follicles, and Bcl-2/Bax expression ratio showed that coadministration of apigenin and BMSCs significantly recovered the ovarian function, structure, and apoptosis compared to the control, BMSC, and apigenin groups (p < 0.001).

**Conclusion:**

The results suggest that the effect of coadministration of apigenin and BMSCs is maybe more effective than the effect of their administrations individually on the recovery of damaged ovaries following the chemotherapy with cyclophosphamide in rats.

## 1. Introduction

Ovary is one of the most susceptible organs against chemotherapy in young girls and women who fight cancer (1). Side effects of chemotherapy can damage ovarian granulosa cells so that folliculogenesis may be impaired (2). Cyclophosphamide is one of the most widely used chemotherapy drugs which causes damage to the ovaries (3). There are several treatment procedures for damaged ovaries such as hormone therapy, ovarian tissue freezing, stem cell therapy, and the use of antioxidants (4). Certainly, hormone therapy is not suitable for cancer patients because it increases the likelihood of cancer recurrence (5). Ovarian tissue freezing is also unfavorable because it requires two surgeries to remove and transfer tissue and the possibility of returning its performance is low (6). Lately, it has been noticed that transplantation of bone marrow stromal cells (BMSCs), a type of mesenchymal stem cells (MSCs), may recover ovarian impair after chemotherapy (7). BMSCs can secrete many growth factors, differentiate into other cell lines, and replace defective cells (8). On the other hand, it has been shown that the use of apigenin as an antioxidant may preserve the damaged ovaries (9). Apigenin is from flavonoids category that is present in some fruits and vegetables with antioxidative, antiapoptotic, and anti-inflammatory properties (10). Apigenin prevents cell apoptosis by suppressing reactive oxygen species (ROS) compounds (11). Moreover, apigenin may promote the differentiation of stem cells (12, 13).

Although the useful impacts of apigenin and BMSCs on damaged ovaries have been studied individually, there is no report yet about the impact of coadministration of them on the improvement of damaged ovaries following chemotherapy. Thus, for the first time, we assessed the impact of coadministration of apigenin and BMSCs on ovarian function, structure, and apoptosis after creating a model of chemotherapy with cyclophosphamide in rat.

## 2. Materials and Methods

### Experimental animals

In this experimental study, 40 adult female Wistar rats (weighing 180–200 gr, 10 wk old) were applied. The rats were kept at a controlled temperature (25±2°C) and had free access to water and food. Vaginal smear samples were obtained daily and only those displayed at least two consecutive normal vaginal estrus cycles were applied in the tests.

### Culture and characterization of BMSCs 

Immediately after killing an adult rat, femurs and tibias were taken out. To kill the rat, it was first anesthetized with an intraperitoneal injection of 80 mg/kg ketamine and 10 mg/kg xylazine and then cervical dislocation was performed. The bone marrow was flushed out with 10 ml of Dulbecco's Modified Eagle Medium (DMEM) + 10% fetal bovine serum (FBS) (Gibco, Germany) in two flasks and incubated in the culture medium containing 10% FBS and 1% penicillin/streptomycin (Gibco, Germany), at 37°C, 95% humidity, and 5% CO${}_{2}$. After 48 hr, the culture medium was replaced. The adherent cells were subcultured four times (14, 15). To analyze the expression of the BMSC surface markers, more than 100,000 cells were incubated to fluorescence-labeled monoclonal antibodies against CD29, CD34, CD44, CD45, and CD90 (Sigma, China). After a 10-min wash with PBS, the labeled cells were analyzed using a flow cytometry apparatus (BD FACS Calibur) (7).

### Creating the chemotherapy model

To damage the ovaries, a chemotherapy model was performed. First, cyclophosphamide (Sigma, China) diluted in normal saline was injected at 50 mg/kg intraperitoneally, then injected at 8 mg/kg daily for 13 consecutive days, in total for 14 days. The percentage of cyclophosphamide dilution was 1% (16).

### Grouping and injection methods in the groups

After creating the model of chemotherapy, the rats were randomly divided into four groups (n = 10/each): (I) Control group, 25 µl of culture medium (DMEM) was directly injected into the bilateral ovaries once; (II) BMSC group, 2 × 10${}^{6}$ BMSCs suspended in 25 µl of DMEM were directly injected into the bilateral ovaries once (17); (III) Apigenin group, 10 mg/kg of apigenin (diluted in DMSO) was injected intraperitoneally for 14 days (18); and (IV) Co-administration of apigenin and BMSC group, injection of apigenin and BMSC were performed together. To inject BMSCs into the bilateral ovaries, the rats were anesthetized and a longitudinal incision about 2 cm was performed below the costovertebral angle on the dorsal midline of the rats to expose the bilateral ovaries. Then, BMSCs or culture medium (DMEM) was injected into the ovaries. Finally, the skin was sutured with 5–0 silk (19).

### Anti-Müllerian hormone evaluation

Four wk after treatment, serum anti-mullerian hormone (AMH) level of the groups was measured by enzyme-linked immunosorbent assay (ELISA) kit (Shanghai Crystal day Biotech, China) in all groups, according to the manufacturer's instruction (20).

### Evaluating the ability of ovulation

Four wk after treatment, the rats were intraperitoneally superovulated by 150 IU/kg of pregnant mare serum gonadotropin (PMSG) (Sigma, China), followed by 75 IU/kg of human chorionic gonadotropin (hCG) (Sigma, China) 48 hr later; about 14–16 hr after the hCG injection, oocytes were collected from the oviduct (7, 21).

### Histological evaluation 

Four wk after treatment, harvested ovaries were fixed in 4% paraformaldehyde. After paraffin embedding, serial sections at 5-µm thickness were prepared. From each ovary, five sections were randomly selected and routine hematoxylin and eosin (H&E) staining was accomplished for histological evaluation with light microscopy. The number of follicles in different stages comprising primordial, primary, secondary, and antral was counted (22, 23).

### Western blot assays

“Four wk after treatment, the ovaries were lysed with RIPA buffer (Cell Signaling Technology, Netherlands) and protease inhibitor (Roche, Switzerland) on ice for 30 min. The mixture was centrifuged for 20 min at 4°C. Then, identical values of proteins (80 µg) were loaded on sodium dodecyl sulfate (SDS) polyacrylamide gel and detached by electrophoresis. After transferring the proteins to nitrocellulose membranes (Amersham Biosciences, USA), the membranes were blocked by 5% skim milk in Tris-buffered saline (TBS) and incubated with primary antibodies for Bax (1:1000), Bcl-2 (1:1000) and β-actin (1:1000) (Abcam, USA) overnight at 4°C. After washing with TBS, 0.1% Tween 20 (TBS-T), the membranes were incubated with secondary antibody conjugated with horseradish peroxidase (HRP). Immunoreactive bands were observed with a chemiluminescence detection system (Amersham Biosciences, USA). Densities of the bands were quantified by densitometric analysis and Image J software. β-actin was used as the internal control to normalize the protein levels" (24).

### Ethical consideration

All procedures were approved by ethical committee of Semnan University of Medical Sciences (Semnan, Iran) and Islamic Azad University (Tehran, Iran). The ethical code is IR.IAU.SRB.REC.1397.136.

### Statistical analysis

After verifying the normality of variances assumptions, data were analyzed using the Statistical Package for Social Sciences (SPSS), version 16/0 (SPSS Inc., Chicago, Illinois, USA) and one-way analysis of variance (ANOVA) and Tukey statistical tests. Mean ± standard error (SE) difference was considered significant at p < 0.05 level.

## 3. Results

### BMSC cultivation and characterization

**Figure 1:**
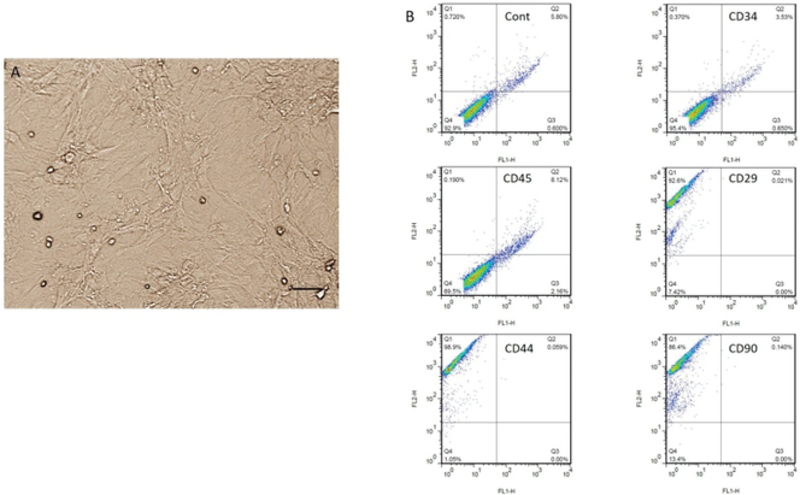
Cultivation and identification of bone marrow stromal cells (BMSCs). (A) Cultured BMSCs at passage 4. (B) The results of flow cytometry show BMSCs are positive for CD29, CD44, and CD90 and negative for CD34 and CD45. Scale bar: 50 µm.

A few days later, the cultured BMSCs were spindle-shaped. After repeating passages, the BMSCs became morphologically homogeneous. Most of the BMSCs expressed stromal cell markers including CD29, CD44, and CD90 while did not express the markers of hematopoietic cells comprising CD34 and CD45 (Figure 1).

### Level of AMH serum

**Figure 2:**
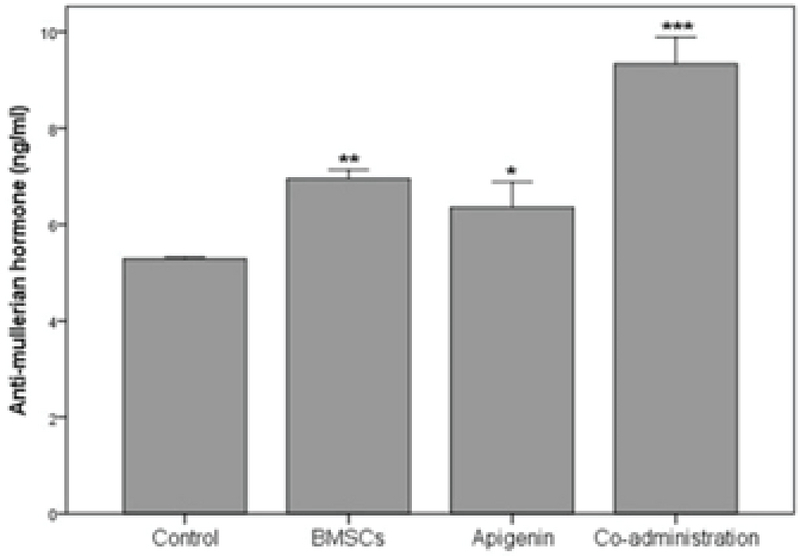
The level of AMH serum in the control and experimental groups four weeks after treatment. *P < 0.05, **P < 0.01, ***P < 0.001 versus control group.

Four weeks after the treatment, the results of the ELISA test demonstrated that the levels of AMH serum in the BMSC group (p = 0.001) and apigenin group (p = 0.02) were significantly higher compared to the control group. Moreover, the results of the coadministration group were significantly higher in comparison with BMSC, apigenin and control groups (p < 0.001) (Figure 2).

### Evaluating the ability of ovulation

**Figure 3:**
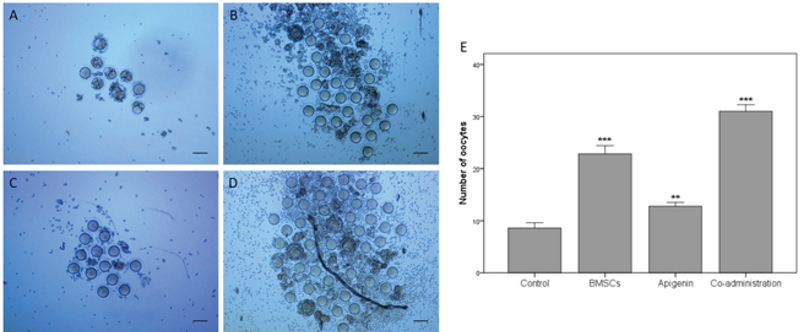
The oocytes were collected from the ovaries following superovulation four weeks after treatment in (A) Control group,** (**B) BMSC group,** (**C) Apigenin group,** (**D) Coadministration of the BMSC and apigenin groups, and (E) The results of the number of oocytes. Scale bars: 100 µm. **P < 0.01, ***P < 0.001 versus control group.

The results of ovulation ability showed that the number of collected oocytes after superovulation in BMSC group (p < 0.001) and apigenin group (p = 0.001) was significantly more than the control group and the results of co-administration group were significantly more than BMSC, apigenin and control groups (p < 0.001; Figure 3).

### Histological evaluation of the ovaries

**Figure 4:**
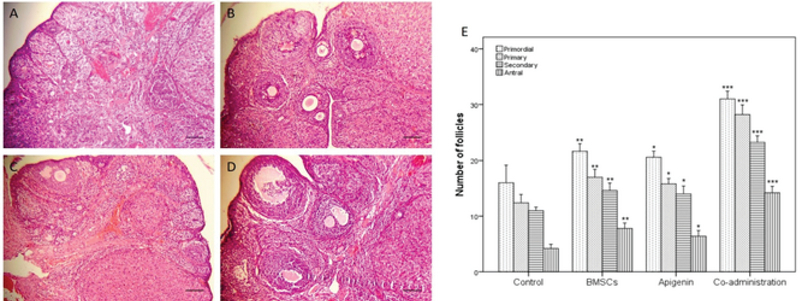
The number of follicles four weeks after treatment. Hematoxylin and eosin (H&E) staining of ovaries in (A) Control group,** (**B) BMSC group,** (**C) Apigenin group,** (**D) Coadministration of the BMSC and apigenin groups, and (E) The results of the number of follicles in different stages. Scale bars: 100 µm. *P < 0.05, **P < 0.01, ***P < 0.001 versus control group.

The results of H&E staining showed that the number of ovarian follicles in different stages in BMSC group (p = 0.002) and apigenin group (p = 0.019) was significantly higher than the control group. The number of follicles in coadministration group was significantly higher than BMSC, apigenin and control groups (p < 0.001, Figure 4).

### Analysis of the Bcl-2 and Bax expression in the ovaries 

**Figure 5:**
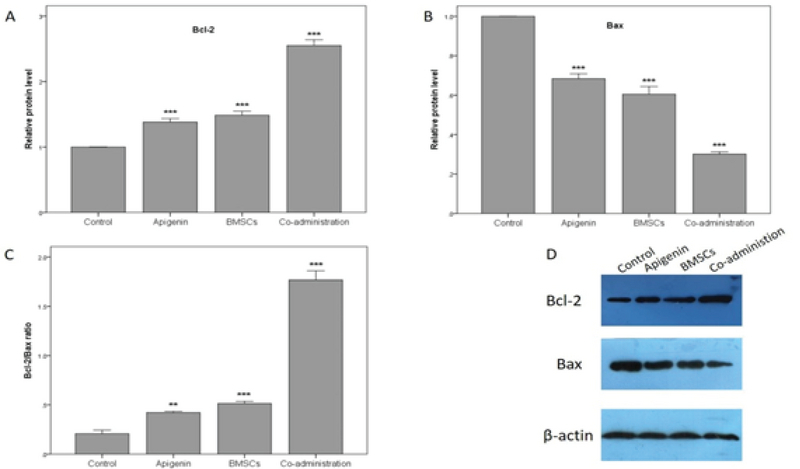
The expression of Bcl-2 and Bax proteins by western blot assay four weeks after treatment. (A) The results of the expression of ovarian Bcl-2 protein. (B) The results of the expression of ovarian Bax protein. (C) The results of the Bcl-2/Bax ratio. (D) Immunoblot of Bcl-2, Bax and β-actin proteins. **P < 0.01, ***P < 0.001 versus control group.

The results of expression of ovarian Bcl-2 and Bax proteins showed that Bcl-2 expression in the experimental groups was significantly higher than the control group (p < 0.001), and in coadministration group, it was significantly higher than the BMSC and apigenin groups (p < 0.001). Bax expression in the experimental groups was significantly lower than the control group (p < 0.001), and in coadministration group, it was significantly lower than the BMSC and apigenin groups (p < 0.001). The Bcl-2/Bax ratio was significantly increased in the coadministration group in comparison with the control, BMSC, and apigenin groups (p < 0.001, Figure 5).

## 4. Discussion 

In the current study, for the first time, we investigated the impact of coadministration of apigenin and BMSCs on damaged ovaries after creating a model of chemotherapy with cyclophosphamide in rats. Overall, the results showed that a coadministration of apigenin and BMSC significantly improved the function, structure, and apoptosis in the damaged ovaries.

We examined the ovaries in terms of function, structure, and apoptosis. To assess ovarian function, the ability of ovulation by superovulation and the level of AMH serum by ELISA kit were evaluated. AMH is a dimeric glycoprotein from transforming growth factor β family which is produced by granulosa cells of preantral follicles. Therefore, when apoptosis occurs in granulosa cells, the amount of AMH decreases. Indeed, AMH is an important indicator for the ovarian follicular content and can be used as a marker for ovarian dysfunction (25). To assess ovarian structure, the number of follicles in different stages were evaluated by H&E staining. Also to assess apoptosis in ovarian granulosa cells, the expression ratio of Bcl-2/Bax proteins was evaluated by western blot because the Bcl-2 and Bax genes are respectively characterized as antiapoptotic and proapoptotic agents (24, 26).

The results of the BMSC group were significantly more desirable than the control group that these results were consistent with other studies (7, 27). BMSCs as a type of MSCs are a suitable candidate for cell therapy in damaged ovaries. Liu and co-workers have reported that MSCs improve tissues chiefly via differentiation and paracrine effects (28). Various studies have demonstrated that BMSCs secrete some growth factors which can recover damaged ovaries and prevent cell apoptosis. Some of these growth factors are insulin-like growth factor 1, vascular endothelial growth factor, basic fibroblast growth factor, and hepatocyte growth factor (7, 27).

On the other hand, the results of apigenin group showed that apigenin as an antioxidant significantly improved the ovaries than the control group. These results consent with Tang's study. Tang and co-workers showed that apigenin could protect ovaries by inhibiting the self-renewal capacity of human ovarian cancer (9). While the results of our study were contrary to Soyman's study, Soyman and co-workers showed that a single dose of 15 mg/kg apigenin has no significant protective effect on ovarian injury following ischemia and reperfusion (29). The reason for this contradiction was probably due to the use of a single dose of apigenin in Soyman's study against the administration of 10 mg/kg apigenin for 14 consecutive days in our study. Some articles have reported that apigenin has protective effects on various cell types. Anusha and co-workers showed that apigenin has protective effects on the rat model of Parkinson's disease by suppressing neuro-inflammation factors and inhibiting apoptosis induced by oxidative stress (18). Zhang and co-workers showed that apigenin has neuroprotective effects on spinal cord injury in rats (30). Liu and co-workers showed that apigenin has a protective role in undifferentiated dental pulp cells (31). Apigenin plays an important role in fatty acid transport and lipid catabolism in mitochondria. Apigenin increases mitochondrial activity and produces ATP by increasing β-oxidation of fatty acids hence providing energy for cellular growth suppressing apoptosis (32). In addition, apigenin can prevent cell apoptosis by repressing ROS compounds (11). Accumulation of ROS in ovarian follicles leads to discharge in the ATP repository, which reduces follicle quality. Antioxidants as ROS scavengers and energy production facilitators may be responsible for beneficial effects on ovarian function and follicular survival (33).

Given the potential effects of apigenin on the differentiation of stem cells, coadministration of apigenin and BMSCs may offer a novel clinical approach to the recovery of damaged ovaries following chemotherapy. The results of the coadministration group were significantly more desirable than BMSC, apigenin, and control groups. The reasons were likely related to the compound of beneficial effects of MSCs and apigenin with various action mechanisms in the recovery of ovaries after chemotherapy. Additionally, apigenin might increase the differentiation of the transplanted BMSCs to replace damaged ovarian cells. Dawn and co-workers reported that the differentiation of adult bone marrow-derived cells into other cells of various organs has been repeatedly confirmed (8). Samet and coworkers have reported that apigenin has the potential synergistic effects on the differentiation of hematopoietic stem cells (13). Zhang and co-workers have shown that apigenin is effective on differentiating MSCs in vitro (12). They have reported that apigenin promotes the osteogenesis of hMSCs by increasing alkaline phosphatase activity and mineralization in hMSCs. Thereby using apigenin and MSCs in the ovary might cause osteogenic differentiation inside the ovaries. In this regard, Mao and co-workers have reported that the two main factors for osteogenic differentiation from MSCs are substrate stiffness and neighboring cells (34). Therefore, due to the lack of substrate stiffness and osteocyte in ovaries, the probability of BMSC differentiation into osteogenic cells was low. Zhang and co-workers in an in vitro study have shown that apigenin inhibits inflammatory factors and at doses < 40 µmol/L have no harmful effect on MSCs. However, at the dose of 80 µmol/L, apigenin significantly increased apoptosis in these cells (35). This issue shows that apigenin as an antioxidant may be harmful if consumed in too large quantities. In the present study, we observed that 10 mg/kg apigenin with BMSC transplantation has favorable effects on the rat ovarian restoration.

In the current study, BMSC transplantation was performed locally in the ovaries while apigenin injection was given intraperitoneally. According to related studies, our hypothesis was that the prescribed models would probably be the most effective mode. Liu and co-workers after comparing the local and systemic administration of MSCs reported that local administration of stem cells is the most efficient way for stem cell homing and differentiation (28). Also, the effectiveness of intraperitoneal injection of apigenin in other tissues has been repeatedly reported (18, 30, 31). While the local injection of apigenin in the ovary has not been done so far.

### Limitation

This study had some limitations. The number of samples was small, so the larger sample size is required. Also, more research is necessary to clarify the molecular mechanisms underlying apigenin and BMSCs function in ovarian repair after chemotherapy. In addition, the effect of different doses of apigenin and the fate of transplanted BMSCs have not been investigated in this study.

## 5. Conclusion

The results suggest that the effect of co-administration of apigenin and BMSCs is maybe more effective than the effect of their administrations individually on the recovery of damaged ovaries following the chemotherapy with cyclophosphamide in rats.

##  Conflict of Interest

There is no conflict of interest in this article.
